# Application of persuasive system design in mobile health interventions for chronic disease management: a mini review

**DOI:** 10.3389/fpubh.2025.1718371

**Published:** 2025-11-20

**Authors:** Kai Zhang, Yurong Jiang, Keyue Xi, Ningna Sun

**Affiliations:** Department of Industrial Design, School of Art, Jiangsu University, Zhenjiang, Jiangsu, China

**Keywords:** persuasive technology, persuasive system design, mHealth, chronic diseases, PSD framework

## Abstract

Persuasive System Design (PSD) has emerged as a pivotal framework in the development of mobile health (mHealth) interventions aimed at chronic disease management. While numerous systems have been designed under its theoretical guidance, the implementation patterns of its design principles and the efficacy of resulting interventions remain inadequately synthesized. This mini-review consolidates recent evidence to elucidate the application characteristics of the PSD framework, revealing a structural imbalance in the adoption of its principles: a predominant focus on primary task and dialogue support, contrasted with a notable underutilization of social support and credibility-enhancing features. Furthermore, we explore the adaptive mechanisms linking PSD principles with specific mHealth functions and technological platforms, highlighting how such synergies can be tailored to diverse clinical contexts. Critical methodological shortcomings are also identified, including an overreliance on physiological outcomes in randomized controlled trials and a general neglect of qualitative insights and health economic evaluations. By identifying these gaps and trends, this review aims to inform the future development of theoretically grounded, persuasive, and sustainable digital health interventions for chronic disease management.

## 1 Introduction

Mobile health (mHealth) refers to the application of wireless technologies, particularly mobile phones and associated platforms, within healthcare delivery systems to enhance patient engagement through mediated interactions. This technological paradigm encompasses diverse platforms such as web-based interfaces, smartphone applications, wearable devices, and other wireless instrumentation (1). Accumulating evidence positions mHealth as an innovative approach for facilitating positive behavioral modifications (2). Given the persistent nature of chronic conditions necessitating longitudinal management, smartphone-delivered interventions offer considerable promise. Specifically, mHealth solutions demonstrate efficacy in augmenting self-management capacities and catalyzing health-related behavior modification (3).

The Persuasive System Design (PSD) framework, initially conceptualized by Oinas-Kukkonen and Harjumaa (4), provides a mini framework for developing and evaluating systems designed to influence user attitudes and behaviors. The framework proposes 28 persuasive system design principles (also referred to as PSD principles), which are categorized into four groups: primary task support, dialogue support, system credibility support, and social support (5). Within the realm of chronic disease management, numerous information systems have been developed under the theoretical guidance of the PSD framework (6). However, critical knowledge gaps persist regarding both the methodological approaches for implementing this model and the subsequent evaluation of intervention efficacy.

## 2 Methods

**Figure 1:**
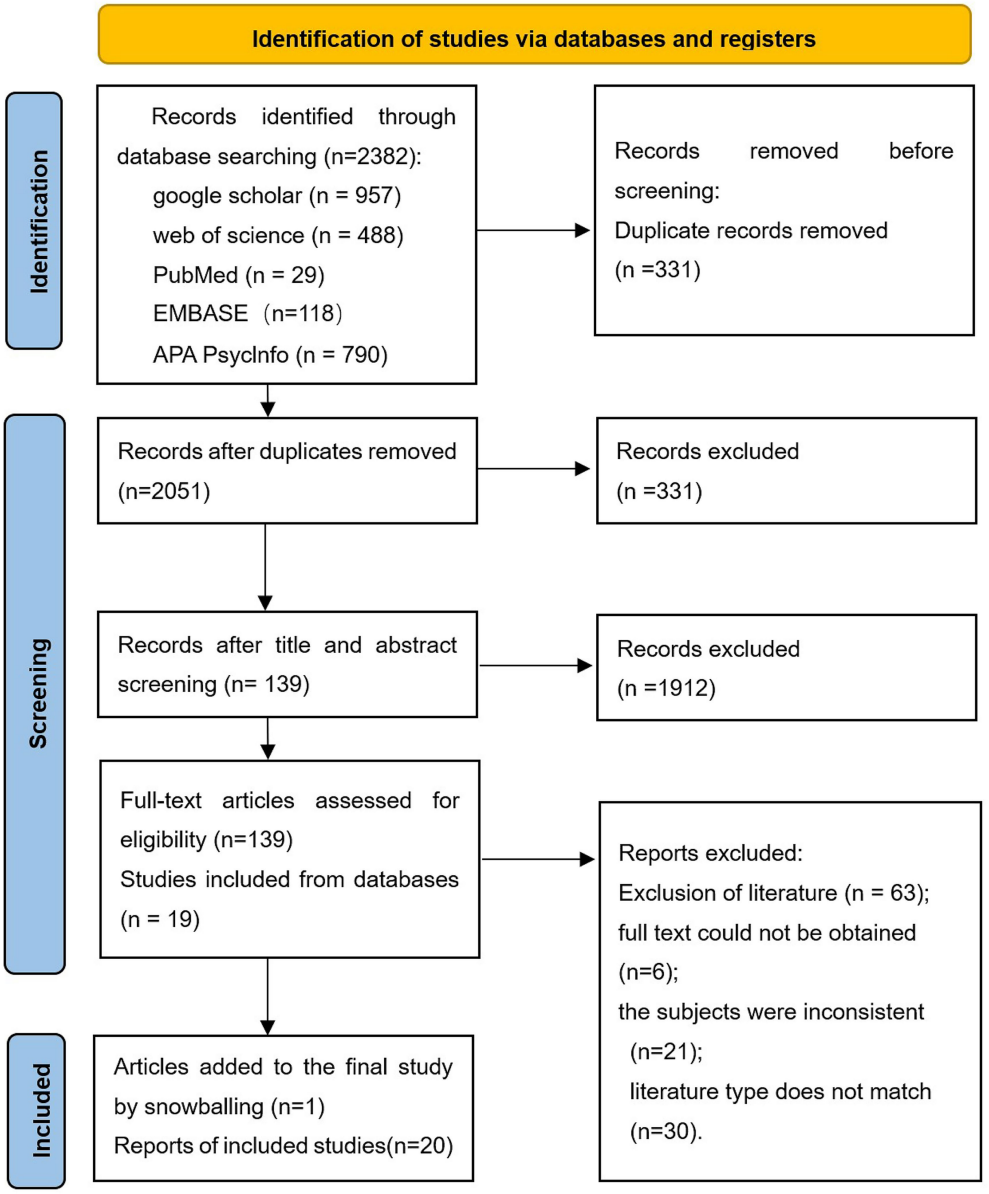
PRISMA flow diagram of study selection. Schemes follow the same formatting.

This mini review was conducted according to the PRISMA 2020 guidelines (7, 8) and registered in PROSPERO. A comprehensive literature search was performed across multiple databases from inception to June 2025. Following a structured screening process, as illustrated in Figures 1, 20 studies meeting predefined inclusion criteria were included for analysis. Detailed procedures are available in the PROSPERO registry.

### 2.1 Research aims

- To evaluate how PSD principles are applied and their structural features by systematically analyzing the distribution and use of the four principles’ categories, thereby uncovering application biases and underlying weaknesses.
- To construct and elucidate a “principle-function-platform” synergistic adaptation framework by identifying core mHealth functions and technological platforms, and mapping their relationships with PSD principles.
- To critically evaluate the current research methodology and evidence system by summarizing dominant research designs, assessment metrics, and their limitations, thereby identifying critical gaps in the existing evidence chain.

### 2.2 Literature screening criteria and quality assessment

The inclusion criteria were as follows: ① the study population consisted of patients with chronic diseases, such as hypertension, diabetes, arthritis, and obesity; ② the study focused on mobile health (mHealth) interventions applying the Persuasive Systems Design (PSD) framework for chronic disease patients; ③ the study designs included randomized controlled trials, cross-sectional studies, qualitative research, among others. The Mixed Methods Appraisal Tool (MMAT), suitable for the economic and health fields, was employed to assess the quality of the included studies (9–11). Each study was independently evaluated by two reviewers. Any discrepancies were resolved through discussion, and when necessary, a third reviewer was consulted for arbitration.

## 3 Results

This mini review ultimately included 20 studies through systematic retrieval and screening. The literature quality analysis revealed that the majority of RCTs had a low-to-moderate risk of bias, while the overall quality of the observational studies was deemed acceptable. The detailed assessment results are provided in Supplementary Table 1. A detailed analysis was conducted on the included characteristics, such as publication year, region, disease condition, sample size, intervention duration, and intervention measures; the comprehensive data are documented in Supplementary Table 2. Analysis of Supplementary Tables 1, 2 yielded the following findings: Temporal analysis showed that the included publications span from 2013 to 2025, with relevant explorations documented throughout this period, as presented in Supplementary Table 1 (12–31). The early- and mid-period studies (2013–2017) were relatively scarce and typically featured smaller sample sizes with shorter follow-up durations. Notably, 2019 emerged as the most intensive research year, with multiple significant publications (20–24).

Geographically, the studies exhibited substantial concentration in Europe and North America, revealing marked regional disparities in research distribution. Finland (12, 13, 25, 26, 31), the Netherlands (15, 23, 28, 30), and the United States (17, 18, 20) accounted for the majority of publications, while Asian and African countries demonstrated comparatively limited research output in this domain (14, 19). This uneven geographical distribution can be attributed in part to the Finnish origins of the PSD framework. It also reflects disparities in the application of digital health technologies based on PSD principles, the allocation of research resources, and considerations for cultural adaptation. Consequently, the generalizability of research findings to non-Western cultural contexts may be limited. Future research should place greater emphasis on the application of PSD within culturally diverse settings.

Disease-specific analysis identified three predominant research focuses: (a) metabolic disorders (8 studies) (12, 13, 17–19, 25, 26, 30), (b) mental health conditions (5 studies) (14, 20, 21, 28, 31), and (c) cardiovascular diseases (4 studies) (15, 22, 24, 29). The depth and breadth of research varied significantly across different disease categories. Future investigations should expand to more geographic regions and disease areas to further validate and refine the universal applicability and effectiveness of persuasive systems in chronic disease management.

### 3.1 PSD-based mHealth applications in chronic disease management: an analysis of principles, functions, and platforms

#### 3.1.1 Application of PSD principles: dominance of instrumental support and deficiency in social support

**Figure 2:**
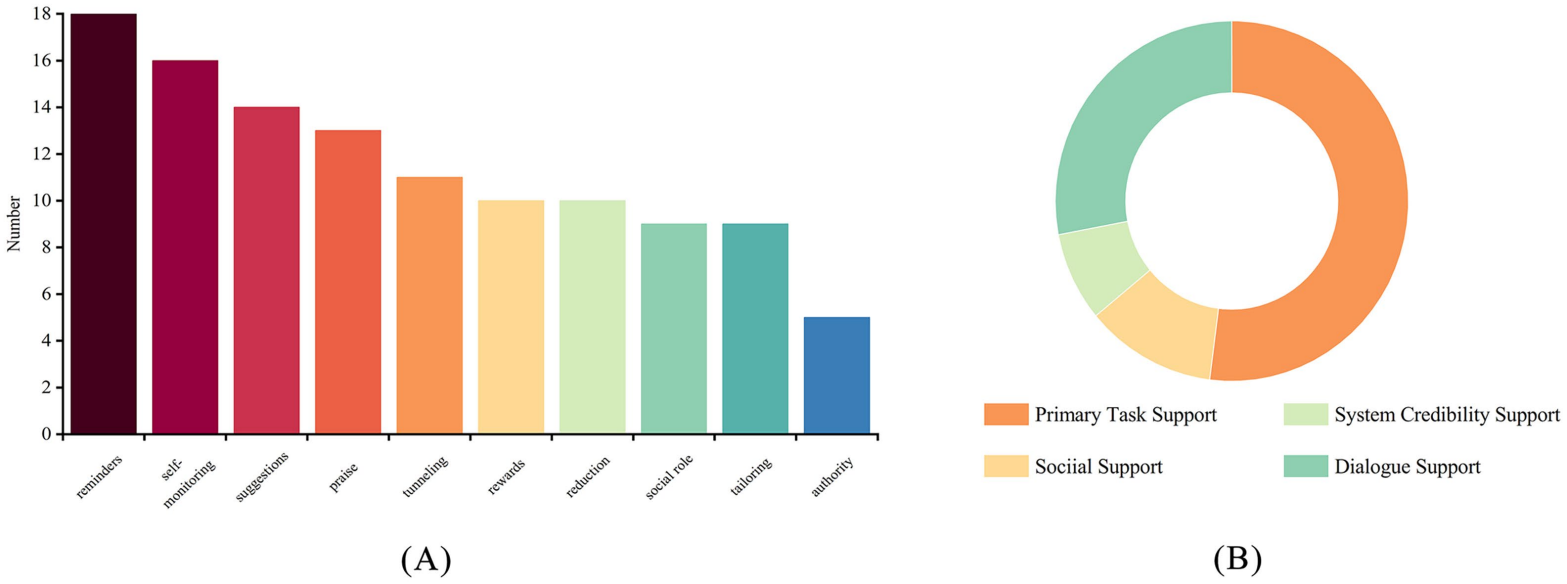
**(A)** The top 10 most frequently applied persuasive system design (PSD) principles across the 20 included studies. **(B)** The four core categories of the persuasive system design (PSD) framework applied in the analysis.

Using the four categories framework introduced by Oinas-Kukkonen as the foundation for discussion, our analysis of the 28 persuasive principles across 20 studies (4) reveals substantial variations in their application. As detailed in Supplementary Table 3, the relative emphasis on different PSD categories is visually summarized in Figure 2A, while Figure 2B highlights the 10 most frequently applied persuasive strategies. Characterized by a dominance of instrumental principles and weak social support.

The principles under the Primary Task Support category were the most extensively implemented. Among them, Self-Monitoring emerged as the most frequently applied principle (*n* = 16, 80%), utilized across diverse chronic disease management contexts including obesity (12, 13, 18) and depression (20, 21)to facilitate health data tracking and behavioral visualization. The Reduction principle ranked second (*n* = 10, 50%), primarily manifested through optimized user interfaces and interaction flows to minimize operational burdens. Conversely, Simulation (14, 15, 22, 24, 27, 28) and Rehearsal (12, 14, 21, 28, 29) principles demonstrated lower adoption frequencies, indicating underutilization in current practice.

Among the principles in the Dialogue Support category, the Reminders principle demonstrated the highest implementation rate (*n* = 18, 90%), predominantly facilitating medication adherence management (15, 23, 26). In stark contrast, the Similarity principle (24, 30) designed to enhance system affinity and persuasiveness through common ground establishment, showed minimal adoption. This indicates insufficient exploration of personalized user features and context replication for trust-building purposes.

Within the System Credibility Support category, these principles exhibited suboptimal application frequencies, revealing critical shortcomings in credibility-establishing design. While Expertise (12, 14, 15, 22, 23, 31) and Authority (15, 18, 19, 22, 23, 27) principles appeared sporadically [e.g., (19) developed a system that provided expert certifications or medical endorsements], their utilization remained generally low. Notably, Surface Credibility was absent, reflecting systematic neglect of visual-interface persuasive design elements.

An analysis of the Social Support category revealed that its principles were markedly underutilized, with application rates for all principles not exceeding 15%. This indicates that social support mechanisms have not been effectively integrated. Social Comparison (16, 22, 31) and Cooperation (15, 16, 31) are the relatively mainstream choices, motivating users by comparing progress or providing collaborative goals. However, Normative Influence (30), which aims to exert influence through group norms, and Recognition (27), which provides public acknowledgment for user achievements, are almost absent. This suggests that current application designs have failed to effectively integrate social mechanisms, remaining almost exclusively within the scope of individual use. Consequently, a critical opportunity to leverage social dynamics for sustained user engagement has been missed (4, 32).

#### 3.1.2 Correlational analysis of PSD framework application in mHealth functions: commonalities and distinctive features across diseases

After consolidating the specific mHealth functions mentioned in the 20 included studies and their corresponding PSD principles into Table 1, we performed a systematic extraction and thematic analysis of the intervention features. This process identified and categorized 13 cross-cutting persuasive functional domains for chronic disease management: ① Goal Management, ② Health Guidelines, ③ Health Reminders, ④ Professional Guidance, ⑤ Data Monitoring, ⑥ Incentive Mechanism, ⑦Personalized Customization, ⑧ Goal Tracking, ⑨ Risk Assessment, ⑩ Community Learning, ⑪ Community Competition, ⑫ Self-Assessment Tool, ⑬ Crisis Intervention, The analytical process followed established principles of qualitative content analysis, ensuring mutual exclusivity and comprehensiveness in functional categorization. These domains constitute a disease-agnostic taxonomy of Persuasive System Design (PSD) framework applications, establishing foundational patterns for subsequent evidence synthesis, as detailed in the “Functions” column of Table 1.

**Table 1:** Summary of function domains, platforms, and PSD principles in the 20 included mHealth interventions.

No.	Included study	Chronic condition(s)	mHealth platform	Primary functions	Functional domains	PSD principles
1	De Oliveira et al. (12)	Obesity	Web	Record weight and BMI progress; Gradually adjust weight loss goals; Provide user reward incentives; Remind of personal goal completion; Display authoritative health support; Minimize system operation interference	① Goal Management; ③ Health Reminders ⑤ Data Monitoring; ⑥ Incentive Mechanism;	tailoring, self-monitoring, reduction, rehearsal, praise, reminders, trustworthiness, expertise
2	Turkkila et al. (13)	Obesity	Web	Provide weekly health information articles; Set personalized weight loss goals; Record diet and exercise data; Track weight change trends; Send login and task reminders; Apply behavioral persuasion principles	① Goal Management; ② Health Guidelines; ③ Health Reminders; ⑤ Data Monitoring; ⑦ Personalized Customization	self-monitoring, reduction, tunneling, tailoring, praise, reminders, suggestion, liking, verifiability, social learning, social facilitation
3	Guracho et al. (14)	Depression and Anxiety Disorders	APP	Provide mental health education information; Conduct self-assessments; Teach relaxation and mindfulness techniques; Set medication and appointment reminders; Provide emergency psychological assistance; Support multi-language interface switching; Ensure user data privacy; Allow user progress feedback; Connect to the doctor consultation function	② Health Guidelines; ③ Health Reminders; ④ Professional Guidance; ⑦ Personalized Customization ⑫ Self-Assessment Tool; ⑬ Crisis Intervention;	reduction, tunneling, tailoring, self-monitoring, simulation, rehearsal, trustworthiness, expertise, real-world feel, third-party endorsements, verifiability, praise, reminders, suggestions, liking, social role, cooperation
4	Bente et al. (15)	Cardiovascular Disease	Web	Record blood pressure and step count data; Set exercise or diet goals; Provide points reward redemption; Remind to record health data; Generate automatic health feedback; Provide lifestyle suggestions; Visualize health data charts; Support online doctor chat; Push personalized suggestions; Set health challenge tasks; Save health history records; Send risk notifications	① Goal Management; ② Health Guidelines; ③ Health Reminders; ④ Professional Guidance; ⑤ Data Monitoring; ⑥ Incentive Mechanism; ⑦ Personalized Customization; ⑧ Goal Tracking; ⑨ Risk Assessment	self-monitoring, tunneling, personalization, tailoring, simulation, rewards, reminders, praise, suggestion, trustworthiness, expertise, competition, cooperation, social role, authority
5	Signorelli et al. (16)	Breast Cancer	APP	Create personalized walking plans; Monitor real-time activity levels; Provide exercise intensity feedback; Guide audio exercise sessions; Adjust plans based on fatigue feedback. Send encouraging messages; Simulate the coach interaction interface; Schedule exercise timetables; Remind daily exercise; View activity history records; Generate weekly progress reports	① Goal Management; ③ Health Reminders ④ Professional Guidance; ⑤ Data Monitoring; ⑥ Incentive Mechanism; ⑦ Personalized Customization; ⑧ Goal Tracking;	tunneling, self-monitoring, suggestion, personalization, praise, social role, reminders
6	Francis et al. (17)	Type 2 Diabetes, Obesity	Wearable Device, App	Send SMS and email reminders; Provide interactive education sessions; Set diet and activity challenges; Track user participation reports; Provide shopping card financial incentives; Conduct motivational interview guidance; Send continuous health tips	① Goal Management; ② Health Guidelines; ③ Health Reminders; ④ Professional Guidance ⑥ Incentive Mechanism; ⑧ Goal Tracking;	reduction, self-monitoring, rewards, reminders, social comparison
7	Yin et al. (18)	Diabetes	Web	Remind to participate in educational activities; Provide diabetes education courses; Set physical activity challenges; Track user interaction data; Provide financial reward mechanism; Conduct motivational goal setting; Send health maintenance SMS	① Goal Management; ② Health Guidelines; ③ Health Reminders; ⑥ Incentive Mechanism ⑧ Goal Tracking;	tunneling, self-monitoring, reduction, reminders, suggestions, rewards, authority
8	Daud et al. (19)	Diabetes, Obesity, etc. (Metabolic Syndrome)	APP, Web	Manage health record information; Assess cardiovascular risk; Set treatment goals; Provide star rating rewards; Record weight and blood glucose data; Provide health coaching guidance; Support doctor remote monitoring; Provide clinical decision support	① Goal Management; ④ Professional Guidance ⑤ Data Monitoring; ⑥ Incentive Mechanism; ⑨ Risk Assessment;	self-monitoring, rewards, suggestions, authority, social role
9	Zhang et al. (20)	Depression/Anxiety	APP	Access mental health content; Set and edit personal goals; Track activity and mood records; Integrate app notification reminders; Provide low-intensity SMS coaching	① Goal Management; ② Health Guidelines; ③ Health Reminders; ④ Professional Guidance ⑧ Goal Tracking;	self-monitoring, reminders
10	Yap et al. (21)	Depression/Anxiety	Web	Generate parenting feedback reports; Recommend interactive parenting modules; Send weekly email reminders; Provide telephone follow-up support; Track module completion status	③ Health Reminders; ④ Professional Guidance ⑦ Personalized Customization; ⑧ Goal Tracking;	tailoring, personalization, tunneling, self-monitoring, reminders, praise, suggestion, social role, rehearsal, expertise
11	Sankaran et al. (22)	Coronary Artery Disease	APP	Record blood pressure and weight data; Remind timely medication; Set personalized exercise goals; Visualize exercise progress; Predict activity contribution value; Provide video coach guidance; Send positive feedback messages; Support doctor remote adjustment	① Goal Management; ③ Health Reminders; ④ Professional Guidance; ⑤ Data Monitoring; ⑥ Incentive Mechanism ⑧ Goal Tracking;	self-monitoring, tailoring, personalization, simulation, reminders, praise, suggestion, expertise, authority, social role
12	Pelle et al. (23)	Knee/Hip Osteoarthritis	APP	Select osteoarthritis goals; Recommend personalized goals; Send daily notifications; Reward achievements; Provide educational information library; Guide exercise practice	① Goal Management; ② Health Guidelines; ③ Health Reminders; ④ Professional Guidance ⑥ Incentive Mechanism; ⑦ Personalized Customization;	self-monitoring, reminders, rewards
13	Coorey et al. (24)	Cardiovascular Disease	Web	Display cardiovascular risk score; Set healthy eating goals; Integrate health record data; Send health prompt messages; Provide anonymous forum support	① Goal Management; ③ Health Reminders; ⑤ Data Monitoring; ⑨ Risk Assessment; ⑩ Community Learning	simulation, reduction, tunneling, self-monitoring, personalization, social comparison, normative influence, praise, rewards, reminders, suggestion, similarity, authority, verifiability, trustworthiness, expertise
14	Teeriniemi et al. (25)	Obesity	Web	Provide healthy lifestyle articles; Allow setting weight loss goals; Record weight and diet data; Send login reminder emails; Apply persuasive design elements; Support mood diary recording	① Goal Management; ② Health Guidelines; ③ Health Reminders; ⑤ Data Monitoring; ⑦ Personalized Customization	reduction, tunneling, tailoring, praise, reminders
15	Klaassen et al. (26)	Diabetes	APP, Web, Wearables	Automatically monitor glucose levels; Set gamified health tasks; Reward points to unlock content; Teach diabetes knowledge games; Provide virtual coach feedback; Support leaderboard competition	① Goal Management; ② Health Guidelines; ④ Professional Guidance; ⑤ Data Monitoring; ⑥ Incentive Mechanism; ⑪ Community Competition	reduction, tunneling, praise, rewards, reminders, suggestions, similarity, social role
16	Karppinen et al. (27)	Diabetes, Obesity, etc.	Web	Record weight and mood data; Track health change trends; Break down complex health tasks; Push customized content weekly; Send login reminders; Provide positive encouragement messages; Optimize user interface experience; Support anonymous experience sharing	③ Health Reminders; ⑤ Data Monitoring; ⑥ Incentive Mechanism; ⑦ Personalized Customization; ⑧ Goal Tracking; ⑩ Community Learning	self-monitoring, reduction, tunneling, tailoring, praise, reminders, suggestions, liking, verifiability, social learning, social facilitation
17	Bartlett et al. (28)	Chronic Obstructive Pulmonary Disease (COPD)	APP	Virtual coach guides walking; Set gradual walking goals; Play exercise encouragement audio; Provide music playback function; Display map activity feedback; Highlight local exercise facilities; Community points reward system; Support competition interaction sharing	① Goal Management; ② Health Guidelines; ④ Professional Guidance; ⑥ Incentive Mechanism; ⑧ Goal Tracking; ⑪ Community Competition	personalization, tunneling, self-monitoring, reminders, praise, suggestion, social role, social learning, social comparison, rewards, recognition, competition, cooperation
18	Kelders et al. (29)	Mild to Moderate Depressive Symptoms	Web	Provide human or automated support; Send motivational text messages; Include multimedia interactive content; Customize success story cases; Allow personalized settings	② Health Guidelines; ④ Professional Guidance; ⑥ Incentive Mechanism; ⑦ Personalized Customization	reminders, praise, suggestion, liking, social role, rehearsal, simulation, tailoring, personalization
19	Salvi et al. (30)	Coronary Artery Disease	Wearable Device, APP, Web	Monitor heart rate and activity data; Provide real-time exercise feedback; Access health education content; Send personalized messages; Remind daily exercise plans; Support doctor-customized training	② Health Guidelines; ③ Health Reminders; ④ Professional Guidance ⑤ Data Monitoring; ⑦ Personalized Customization; ⑧ Goal Tracking;	self-monitoring, rehearsal, rewards, reminders, suggestions, trustworthiness
20	Ahtinen et al. (31)	Stress, Anxiety, Depression, and related psychological issues	APP	Provide structured stress modules; Guide mindful breathing exercises; Reward virtual progress badges; Support diary reflection records; Display recommended navigation steps	② Health Guidelines; ④ Professional Guidance; ⑥ Incentive Mechanism; ⑦ Personalized Customization ⑫ Self-Assessment Tool;	Tunneling, Reduction, Trustworthiness, Rewards, Expertise

The analysis revealed that the functional design of chronic disease management mobile health (mHealth) applications demonstrates both cross-disease foundational features and distinct variations in depth and complexity, attributable to disease-specific pathophysiological mechanisms, management objectives, and patient needs. When contextualized with the distribution patterns of Persuasive System Design (PSD) principles, these variations elucidate underlying behavioral modification intentions embedded in functional implementations.

An examination of the core foundational functions for chronic disease management reveals that, despite disease heterogeneity, they demonstrate remarkable functional convergence in chronic disease management. Three core functionalities constitute the fundamental matrix across interventions: “Goal Management-Health Reminders-and Data Monitoring” (33, 34). These elements align with established behavioral change frameworks, specifically corresponding to the three components of the Fogg Behavior Model (motivation, ability, and prompt) (35). On the basis of setting goals, the mechanistic rationale for this convergence lies in addressing key challenges of long-term disease management. External cueing (36) counters motivational erosion over time. Continuous self-feedback (33) maintains behavioral awareness. Achievable phased goals sustain engagement and adherence (34, 37).

From the perspective of disease-specific functional complexities and PSD integration, the functional architecture of mHealth interventions exhibits progressive sophistication when addressing multifactorial chronic conditions [e.g., cardiovascular diseases (15, 22, 24, 29), musculoskeletal disorders (23)], demonstrating deeper integration with Persuasive System Design (PSD) principles.

The depth of PSD integration manifests in intervention models where principles and functionalities are meticulously matched to core disease management needs.

- For instance, in musculoskeletal disorders (23), the “Professional Guidance” function—incorporating real-time exercise feedback and physician-customized training—deeply embeds the Authority and Trustworthiness principles of PSD. This design leverages medical expertise to enhance scientific rigor and credibility, ultimately improving patient adherence to rehabilitation protocols (4, 22, 38).
- Similarly, in cardiovascular and cerebrovascular interventions (12, 19, 21, 26)functions such as Risk Assessment and Data Monitoring are consistently incorporated. This design facilitates continuous surveillance and early warning for high-risk conditions, forming a robust association with the PSD principles of Self-Monitoring, Suggestion, and Authority.
- For managing psychological conditions such as stress and anxiety (14, 31), the Self-Assessment Tool function is typically added to enable self-evaluation of mental stress. The intervention for these conditions is commonly materialized through PSD principles like Tunneling and Trustworthiness.

The aforementioned examples from various disease conditions exemplify a deeper integration with PSD principles. This evolution reflects a paradigm shift from “generic self-management” to “disease-specific behavioral prescriptions.”

#### 3.1.3 Platform selection strategy: synergistic mapping of principles, functions, and platforms

An analysis of the synergistic mapping patterns between principles, functions, and platforms indicates that the application of PSD principles is not isolated but rather co-mapped with the characteristics of specific functions and technological platforms, forming regular adaptation patterns:

- Mobile Apps, given their high interactivity and immediacy, predominantly integrate Simulation and Rewards principles into Professional Guidance and Incentive Mechanisms. These features suit scenarios requiring frequent engagement and real-time feedback (e.g., psychological interventions, medication adherence training).
- Web, excel in complex data synthesis and long-term planning, prioritizing Self-Monitoring and Tunneling to support Data Monitoring and Health Guidelines. This serves management plans needing macro-level insights and in-depth interaction.
- Wearable Devices, as passive data collectors, focus on continuous physiological tracking. While foundational for Self-Monitoring by supplying objective behavioral data streams, their stand-alone persuasive capabilities remain limited.

This study’s systematic analysis demonstrates that the design of digital health interventions for chronic disease management is a process of systematically adapting clinical needs, patient needs, the PSD framework, and the characteristics of technological platforms. Future intervention designs should move beyond the simple accumulation of functions. Instead, they should consciously combine principles, functions, and platforms based on the characteristics of the target behaviors and the patient’s stage of behavior change, thereby developing digital therapeutics with stronger theoretical underpinnings and greater efficacy.

### 3.2 Methodological approaches and evaluation metrics

The analysis of 20 studies presented in Table 2 reveals that a methodological distinction in current evidence, with Randomized Controlled Trials (RCTs) primarily validating efficacy and observational studies delving into user behavior and design optimization. While complementary, they demonstrate a differential emphasis in their research focus (12–31). A key finding is the prevalent focus on the outcomes of implemented functions (e.g., changes in physiological indicators) by most studies, with a relative neglect of the medium through which these outcomes are achieved—namely, the design quality of the system’s interactive interface and its profound impact on user experience.

**Table 2:** Characteristics of the 20 included studies: chronic disease types, study designs, research methods, and outcome measures.

No.	Included study	Chronic disease type(s)	Study type	Research methods	Outcome measures
1	De Oliveira et al. (12)	Obesity	RCT		a-1 Physiological indicators, b-1 System persuasiveness
2	Turkkila et al. (13)	Obesity	RCT		a-1 Physiological indicators, c-1 Medication/Exercise adherence
6	Francis et al. (17)	Diabetes, Obesity	RCT		b-2 User satisfaction, b-3 System usability, c-1 Medication/Exercise adherence
7	Yin et al. (18)	Diabetes	RCT	Focus group	a-1 Physiological indicators, b-2 User satisfaction, c-1 Medication/Exercise adherence
8	Daud et al. (19)	Metabolic syndrome (e.g., Diabetes, Obesity)	RCT		a-1 Physiological indicators, b-2 User satisfaction, c-1 Medication/Exercise adherence
9	Zhang et al. (20)	Depression, Anxiety	RCT		a-2 Mental health indicators, c-1 Medication/Exercise adherence
10	Yap et al. (21)	Depression, Anxiety	RCT		a-2 Mental health indicators, c-1 Medication/Exercise adherence
11	Sankaran et al. (22)	Coronary Artery Disease	RCT	Semi-structured interviews, Contextual sentiment analysis	a-1 Physiological indicators, b-2 User satisfaction, c-1 Medication/Exercise adherence, c-2 Health-related quality of life
12	Pelle et al. (23)	Knee/Hip Osteoarthritis	RCT	Usability testing, Cost-utility and cost-effectiveness analysis	c-1 Medication/Exercise adherence, c-2 Health-related quality of life, b-5 Number of medical visits, b-6 Treatment costs, b-3 System usability
13	Coorey et al. (24)	Cardiovascular Disease	RCT	Focus group	c-1 Medication/Exercise adherence, c-2 Health-related quality of life, c-4 Illness perception, b-3 System usability, b-4 System credibility
14	Teeriniemi et al. (25)	Obesity	RCT		a-1 Physiological indicators, c-1 Medication/Exercise adherence
18	Kelders et al. (29)	Depression	RCT		a-2 Mental health indicators, b-2 User satisfaction, c-1 Medication/Exercise adherence
19	Salvi et al. (30)	Coronary Artery Disease, Post-MI Rehabilitation	RCT	Semi-structured interviews, Focus group	b-2 User satisfaction, c-1 Medication/Exercise adherence, c-4 Illness perception
3	Guracho et al. (14)	Depression, Anxiety	Analytical study—cross-sectional survey	Focus group, Cognitive walkthrough	b-3 System usability, c-1 Medication/Exercise adherence, a-2 Mental health indicators, b-2 User satisfaction
4	Bente et al. (15)	Cardiovascular Disease	Observational study—descriptive study	Semi-structured interviews, Think-aloud protocol, Usability testing, Log analysis	b-3 System usability, b-2 User satisfaction
5	Signorelli et al. (16)	Breast Cancer	Observational study—descriptive study	Semi-structured interviews, Usability testing, Log analysis	b-3 System usability, b-2 User satisfaction
15	Klaassen et al. (26)	Diabetes	Observational study—descriptive study	Semi-structured interviews, Focus group, Usability testing	c-1 Medication/Exercise adherence, a-1 Physiological indicators, b-2 User satisfaction, c-4 Illness perception
16	Karppinen et al. (27)	Metabolic syndrome (e.g., Diabetes, Obesity)	Observational study—descriptive study	Semi-structured interviews	a-1 Physiological indicators, c-1 Medication/Exercise adherence, b-3 System usability
17	Bartlett et al. (28)	Chronic Obstructive Pulmonary Disease (COPD)	Observational study—descriptive study	Semi-structured interviews, Framework analysis	b-1 System persuasiveness, b-2 User satisfaction
20	Ahtinen et al. (31)	Psychological issues (e.g., Depression, Anxiety)	Observational study—descriptive study	Semi-structured interviews, Thematic coding	a-2 Mental health indicators, b-2 User satisfaction

#### 3.2.1 Dominance and limitations of quantitative evaluation in RCTs

A total of 13 studies (57.1%) employed RCT designs (12, 13, 17–25, 29, 30), offering high-level evidence for the causal efficacy of mHealth interventions on physiological indicators through comparative analysis with control groups (12). However, the evaluation dimensions in these studies present certain limitations and could be enhanced in the following aspects:

- Biomedical Focus of Evaluation Dimensions: 75% of RCTs (*n* = 9) relied predominantly on biomedical indicators (e.g., HbA1c, BMI) or standardized scales, neglecting in-depth exploration of behavioral change mechanisms. For instance, seven studies on metabolic diseases reported anthropometric measures, but only three (18, 22, 24) attempted to investigate the psychological or behavioral drivers behind these changes through focus groups or interviews.
- Assessment of Persuasive System Attributes: The evaluation of key system attributes in Randomized Controlled Trials (RCTs) has been more limited than ideal, despite their importance in the PSD framework. Merely three studies (17, 23, 24) have measured system usability, credibility, or comparable properties as primary endpoints. With most relying on simplified scales like the System Usability Scale (SUS). Crucially, these studies lacked deeper analytical methods—such as usability testing or cognitive walkthroughs—to scrutinize interaction pain points or the efficacy of persuasive logic. Such methodologies are indispensable for rigorous validation of the PSD framework (4).

#### 3.2.2 Recommendation

The Qualitative Merits and Quantitative Constraints in Observational and Analytical StudiesNine studies (eight observational and one analytical) (14–16, 26–28, 31) address the aforementioned gaps by employing a combination of multi-dimensional qualitative methods to focus on user experience. These studies offer critical insights into the user-system interaction logic, providing essential pathways for design optimization:

- Methodological pluralism was evident across the studies, which integrated techniques such as semi-structured interviews (15, 16, 26–28, 31), usability testing (15, 16, 26), log analysis (15, 16), focus groups (14, 26), and cognitive walkthroughs (11) to assess patient needs and adherence. Furthermore, recent work has incorporated more refined techniques for qualitative inquiry, such as thematic coding (31) and framework analysis (28), to deepen the analysis of patient-reported requirements.
- Through mixed methods, these studies were able to more effectively identify the relationships among user research, interface design, and persuasive outcomes. For example:
- Bente et al. (15) revealed through semi-structured interviews that health management platforms can support users in adopting and maintaining healthy lifestyles by optimizing interface design, enabling them to be motivated and reminded of their personal goals.
- Guracho et al. (14), through cognitive walkthroughs of interface design prototypes, found that applications could better meet patient needs and achieve higher credibility.
- An Emphasis on Subjective Metrics in Evaluating Persuasive mHealth Systems: Analysis of outcome measures indicates that eight studies effectively utilized qualitative research endpoints, providing a more holistic assessment of the mHealth platforms themselves. Specifically, seven studies incorporated user satisfaction as an outcome measure (14–16, 26, 28, 31), while four studies employed subjective metrics such as system usability (14–16, 25, 27) and four utilized adherence scales (14, 26, 27, 30). In comparison, the use of objective physiological indicators (15, 16), remained more limited, and health economic analyses represent an area that would benefit from further research development.

These findings directly validate that user research and interface design within the PSD framework can enhance patient adherence. They also highlight the potential value of overlooked PSD principles such as Surface Credibility and Social Support (e.g., the social training needs of COPD patients).

Pervasive Absence of Health Economic Evaluations: Only one study (23) reported a cost-utility analysis. The remaining research did not assess healthcare resource utilization (e.g., number of clinic visits) or cost-effectiveness, overlooking the economic sustainability of interventions. From a modeling perspective, economic evaluation is fundamental to the sustainable development of persuasive systems.

The current research paradigm demonstrates a split: RCTs validate “whether it works” (efficacy), while observational studies explore “why and how it works” (mechanism of action). The standardized design of RCTs often struggles to fully capture the complexity of user experience, while observational studies generally do not establish causal relationships for quantitative physiological or clinical outcomes.

Ideally, mHealth research should integrate the strengths of both approaches. Analysis indicates that interactive interface design serves as the core bridge connecting “functional achievement” and “user,” with its quality directly modulating the effectiveness of PSD framework principles (14–17). Future research should advocate for mixed-methods designs, embedding in-depth, interface-focused qualitative evaluations (such as usability testing, Focus groups, among others) within the RCT framework. This integrated approach would simultaneously assess the clinical effectiveness, economic efficiency, user acceptance, and behavioral change mechanisms of interventions, thereby constructing a comprehensive, multi-dimensional evidence base rooted in the PSD framework and ultimately driving the development of more persuasive and sustainable digital health interventions (23, 24, 30).

## 4 Discussion

### 4.1 Structural imbalance in PSD principle application

The application of PSD principles is characterized by a dominance of instrumental principles and a weakness in social support. Analysis indicates that principles supporting the primary task and dialogue are widely adopted. These directly address core chronic disease management needs—behavior recording, task simplification, timely reminders, and immediate incentives—effectively reducing user cognitive load and initiation barriers. They form the foundation for enhancing instrumental usability and short-term adherence. However, the severe underutilization of social support principles and the general neglect of system credibility support reveal deficiencies in current mHealth designs concerning the cultivation of trust, a sense of belonging, and the leverage of peer influence (39, 40). Chronic disease management is intrinsically a psychosocial process. The absence of effective social support and trustworthy interface experiences may limit the long-term efficacy and user engagement of interventions (41).

### 4.2 Function-principle-platform adaptation mechanism in digital health interventions for chronic diseases

Regarding the “function-principle” relationship, common foundational functions align with PSD principles. Goal-setting, health reminders, and self-monitoring constitute a cross-disease “universal functional module,” supporting common behavior changes in chronic disease management through external prompts, self-feedback, and progressive goal setting (42). The shift towards disease-specific design necessitates an upgrade from “universal self-management” to a “disease-specific behavioral prescription” (43). For complex chronic conditions (e.g., cardiovascular, musculoskeletal diseases), the adaptation of differentiated PSD principles is required: employing authority and personalization principles to enhance disease cognition and self-efficacy, or utilizing tunneling principles to construct structured, low-cognitive-load processes that accommodate the multiple behavioral change needs of individuals with comorbidities.

Analyzing the “Function-Principle-Platform “synergy reveals that different technological platforms necessitate corresponding PSD principles and functions (44). Mobile apps, with their high interactivity and immediacy, are better suited for integrating high-feedback principles (e.g., rewards, simulation) to support real-time behavioral adjustments. Web platforms, adept at complex data integration and long-term goal management, align well with self-monitoring requirements (45). Wearable devices provide objective, continuous data, serving as a foundation for upstream principles. The key implication is that the platform should be selected during intervention development rather than simply porting functions (46).

### 4.3 Limitations and future directions in research and evaluation methods

RCT Dominance with Singular Evaluation Dimensions: While RCTs provide high-level evidence for intervention efficacy, they exhibit an over-reliance on quantitative metrics, particularly physiological indicators. Only a minority integrates qualitative methods, such as focus groups, to explore user experience and behavior change mechanisms.

Observational Studies Provide Depth in Behavioral Understanding but Are Limited in Quantitative Validation: Observational studies (40%), employing diverse qualitative methodologies (semi-structured interviews, focus groups, usability testing, log analysis, cognitive walkthroughs, framework analysis), delve deeply into user needs, pain points (e.g., app abandonment due to interface complexity), and the mechanisms by which PSD designs influence behavior change (e.g., social needs of COPD patients). These findings are crucial for design optimization. However, due to their non-experimental nature, they struggle to establish causal relationships between PSD principles and health outcomes.

Marginalization of mHealth System Attribute Evaluation: While the evaluation of mHealth system attributes themselves (feasibility, usability, user satisfaction, credibility, perceived persuasiveness) appears in some studies, the depth is often insufficient, relying on simplified scales like SUS. Moreover, analyses correlating these attributes with health outcomes are infrequent.

Furthermore, the severe lack of health economic evaluations (e.g., consultation frequency, treatment costs) significantly limits the assessment of intervention cost-effectiveness and decisions regarding scalability.

### 4.4 Limitations and future research directions

Our review has several limitations that should be considered. Firstly, the majority of included studies were conducted in Western countries such as the United States, Finland, and the Netherlands, which may limit the generalizability of our findings across diverse cultural contexts. Furthermore, the general absence of economic evaluations prevented a comprehensive health economic synthesis of the interventions.

Future research should aim to expand empirical investigations to other geographical regions and strengthen the systematic exploration of social support mechanisms. As research on chronic conditions continues to diversify, applying the PSD model to a broader range of chronic diseases would help verify its generalizability. Additionally, future intervention studies could benefit from incorporating cost-effectiveness analyses, thereby contributing to a more complete evidence base for developing theoretically grounded, economically viable, and sustainable digital health solutions.

## 5 Conclusion

This review has identified a pronounced imbalance in the application of PSD principles within chronic disease mHealth interventions, characterized by heavy reliance on instrumental support features (primary task and dialogue support) with corresponding underutilization of social support and credibility-enhancing elements. To maximize intervention effectiveness, future development should pursue strategic “function-principle-platform” alignment to create precisely tailored behavioral prescriptions for specific patient populations and clinical contexts. Methodologically, integrating qualitative insights and health economic evaluations within RCT frameworks would help establish more comprehensive evidence regarding intervention mechanisms, effectiveness, and economic viability. Advancing toward more socially integrated, precisely targeted, and evidence-informed mHealth solutions represents an essential direction for realizing the full potential of digital health in chronic disease management.
