# Supplementary material for: Application of persuasive system design in mobile health interventions for chronic disease management: a mini review
Source: Front Public Health. 2025 Nov 20;13:1718371. doi: 10.3389/fpubh.2025.1718371 (PMC12675468; doi:10.3389/fpubh.2025.1718371)
Supplement: Supplementary file 1 [file Table_1.docx]

Supplementary Material

# Supplementary Tables

**Supplementary Table 1.** **Mixed Methods Appraisal Tool (MMAT) Study Quality Assessment Table for 20 Literatures: Covering Screening Questions and Five Categories of Studies**

|  | **SCREENING QUESTIONS** | | **1. QUALITATIVE STUDIES** | | | | | **2. RANDOMIZED CONTROLLED TRIALS** | | | | | **3. NON-RANDOMIZED STUDIES** | | | | | **4. QUANTITATIVE DESCRIPTIVE STUDIES** | | | | | **5. MIXED METHODS STUDIES** | | | | |
| --- | --- | --- | --- | --- | --- | --- | --- | --- | --- | --- | --- | --- | --- | --- | --- | --- | --- | --- | --- | --- | --- | --- | --- | --- | --- | --- | --- |
| First author | S1. | S2. | 1.1. | 1.2. | 1.3. | 1.4. | 1.5. | 2.1. | 2.2. | 2.3. | 2.4. | 2.5 | 3.1. | 3.2. | 3.3. | 3.4. | 3.5. | 4.1. | 4.2. | 4.3. | 4.4. | 4.5. | 5.1. | 5.2. | 5.3. | 5.4. | 5.5. |
| 1.Coorey | Yes | Yes | Yes | Yes | Yes | Yes | Can't tell | Can't tell | Yes | Yes | No | Can't tell |  |  |  |  |  |  |  |  |  |  | Yes | Yes | Yes | Can't tell | Can't tell |
| 2.Daud | Yes | Yes |  |  |  |  |  | Yes | Yes | Yes | No | Yes |  |  |  |  |  |  |  |  |  |  |  |  |  |  |  |
| 3.de Oliveira | Yes | Yes |  |  |  |  |  | Can't tell | Yes | Yes | No | Yes |  |  |  |  |  |  |  |  |  |  |  |  |  |  |  |
| 4.Francis | Yes | Yes |  |  |  |  |  | Yes | Yes | Can't tell | No | Yes |  |  |  |  |  |  |  |  |  |  |  |  |  |  |  |
| 5.Guracho | Yes | Yes | Yes | Yes | Yes | Yes | Yes |  |  |  |  |  |  |  |  |  |  | Yes | Yes | Yes | Yes | Yes | Yes | Yes | Yes | Can't tell | Can't tell |
| 6.Pelle | Yes | Yes |  |  |  |  |  | Can't tell | Yes | Yes | No | Yes |  |  |  |  |  |  |  |  |  |  |  |  |  |  |  |
| 7.Salvi | Yes | Yes |  |  |  |  |  | Can't tell | Yes | Yes | No | Yes |  |  |  |  |  |  |  |  |  |  |  |  |  |  |  |
| 8.Sankaran | Yes | Yes | Yes | Yes | Yes | Yes | Yes | Yes | Yes | No | No | Yes |  |  |  |  |  |  |  |  |  |  |  |  |  |  |  |
| 9..Teeriniem | Yes | Yes |  |  |  |  |  | Yes | Yes | No | No | Yes |  |  |  |  |  |  |  |  |  |  |  |  |  |  |  |
| 10.Turkkila | Yes | Yes |  |  |  |  |  | Yes | Yes | No | No | Yes |  |  |  |  |  |  |  |  |  |  |  |  |  |  |  |
| 11.Yap | Yes | Yes |  |  |  |  |  | Can't tell | Yes | No | No | Yes |  |  |  |  |  |  |  |  |  |  |  |  |  |  |  |
| 12.Yin | Yes | Yes |  |  |  |  |  | Can't tell | Yes | No | No | No |  |  |  |  |  |  |  |  |  |  |  |  |  |  |  |
| 13.Zhang | Yes | Yes | Yes | Yes | Yes | Yes | Yes |  |  |  |  |  | Yes | Yes | Yes | Yes | Can't tell |  |  |  |  |  | Yes | Yes | Yes | Can't tell | Yes |
| 14.Aino Ahtinen | Yes | Yes | Yes | Yes | Yes | Yes | Can't tell |  |  |  |  |  |  |  |  |  |  | Yes | No | Yes | Can't tell | Yes | Can't tell | Yes | Can't tell | Can't tell | Can't tell |
| 15.Guracho | Yes | Yes | Yes | Yes | Can't tell | Can't tell | Can't tell |  |  |  |  |  |  |  |  |  |  | Can't tell | Can't tell | Yes | Can't tell | Yes | Yes | Yes | Yes | Can't tell | Can't tell |
| 16.Bartlett | Yes | Yes | Yes | Yes | Yes | Yes | Can't tell |  |  |  |  |  |  |  |  |  |  | Yes | Can't tell | Yes | Can't tell | Yes | Yes | Yes | Yes | Can't tell | Can't tell |
| 17.Bente | Yes | Yes | Yes | Yes | Yes | Yes | Yes |  |  |  |  |  |  |  |  |  |  | Yes | Can't tell | Yes | Can't tell | Yes | Yes | Yes | Yes | Can't tell | Can't tell |
| 18.Karppinen | Yes | Yes | Yes | Yes | Yes | Yes | Can't tell |  |  |  |  |  |  |  |  |  |  |  |  |  |  |  |  |  |  |  |  |
| 19.Klaassen | Yes | Yes | Yes | Yes | Yes | Can't tell | Yes |  |  |  |  |  |  |  |  |  |  | Yes | Yes | Yes | Can't tell | Yes | Yes | Yes | Yes | Can't tell | Can't tell |
| 20.Signorelli | Yes | Yes | Yes | Yes | Yes | Yes | Can't tell |  |  |  |  |  |  |  |  |  |  | Can't tell | No | Yes | Can't tell | Yes | Yes | Yes | Yes | Can't tell | Can't tell |

S1. Are there clear research questions? S2. Do the collected data allow to address the research questions?

1.1. Is the qualitative approach appropriate to answer the research question? 1.2. Are the qualitative data collection methods adequate to address the research question?

1.3. Are the findings adequately derived from the data? 1.4. Is the interpretation of results sufficiently substantiated by data?

1.5. Is there coherence between qualitative data sources, collection, analysis and interpretation? 2.1. Is randomization appropriately performed?

2.2. Are the groups comparable at baseline? 2.3. Are there complete outcome data?

2.4. Are outcome assessors blinded to the intervention provided? 2.5 Did the participants adhere to the assigned intervention?

3.1. Are the participants representative of the target population? 3.2. Are measurements appropriate regarding both the outcome and intervention (or exposure)?

3.3. Are there complete outcome data? 3.4. Are the confounders accounted for in the design and analysis?

3.5. During the study period, is the intervention administered (or exposure occurred) as intended? 4.1. Is the sampling strategy relevant to address the research question?

4.2. Is the sample representative of the target population? 4.3. Are the measurements appropriate?

4.4. Is the risk of nonresponse bias low? 4.5. Is the statistical analysis appropriate to answer the research question?

5.1. Is there an adequate rationale for using a mixed methods design to address the research question? 5.2. Are the different components of the study effectively integrated to answer the research question?

5.3. Are the outputs of the integration of qualitative and quantitative components adequately interpreted? 5.4. Are divergences and inconsistencies between quantitative and qualitative results adequately addressed?

**Supplementary Table 2.** Characteristic s of the 20 included studies on persuasive system design in chronic disease mHealth interventions.

| **No.** | **Included Study** | **Year** | **Country** | **Chronic Condition(s)** | **mHealth Type** | **Sample Size** | **Follow-up Duration** | **Follow-up Frequency** | **Study Content / Focus** |
| --- | --- | --- | --- | --- | --- | --- | --- | --- | --- |
| 1 | De Oliveira et al.(9) | 2025 | Finland | Obesity | Web | 96 | 78 weeks | Baseline, 52w, 78w | Investigated the impact of PSD features, design assumptions, and behavioral characteristics on BMI reduction after 6 months of using an mHealth Behavior Change Support System (mHBCSS). |
| 2 | Turkkila et al.(10) | 2025 | Finland | Obesity | Web | 532 | 260 weeks | Baseline, 52w, 104w, 260w | Evaluated the long-term effectiveness of a web-based digital Health Behavior Change Support System (HBCSS) for weight maintenance over a 5-year follow-up. |
| 3 | Guracho et al.(11) | 2025 | Ethiopia | Depression, Anxiety Disorders | APP | 60 | 2 weeks | Not Mentioned | Used Design Science Research Methodology (DSRM) to develop a culturally adapted mental health app integrating PSD principles, assessing functionality usability and user satisfaction. |
| 4 | Bente et al.(12) | 2023 | Netherlands | Cardiovascular Disease | Web | 506 | 26 weeks | Baseline, 26w | Used a mixed-methods design to investigate the supportive role and changing needs of the Vital10PHP platform for health self-management in CVD patients after cardiac rehabilitation. |
| 5 | Signorelli et al.(13) | 2022 | Ireland | Breast Cancer | APP | 4 | 2 weeks | Baseline, 2w | Used the Theoretical Framework of Acceptability (TFA) to develop and evaluate the acceptability and user experience of a personalized walking coach app based on behavior change theory for breast cancer survivors. |
| 6 | Francis et al.(14) | 2021 | USA | Diabetes, Obesity | Wearable Device, App | 388 | 24 weeks | Baseline, 24w | Based on behavior change and gamification theory, evaluated the impact of the "MapTrek" gamified walking intervention on daily step count in adults with obesity/pre-diabetes. |
| 7 | Yin et al.(15) | 2020 | USA | Diabetes | Web | 62 | 12 weeks | Baseline, 6w, 12w | Used mixed methods to develop and evaluate the feasibility and acceptability of a 6-week mHealth diabetes education program for rural vulnerable residents. |
| 8 | Daud et al.(16) | 2020 | Malaysia | Metabolic Syndrome (e.g., Diabetes, Obesity) | APP, Web | 232 | 24 weeks | Baseline, 12w, 24w | Based on the Chronic Care Model (CCM) and persuasive technology theory, developed and evaluated the EMPOWER-SUSTAIN multi-component mHealth intervention on patient activation and self-management behaviors in metabolic syndrome patients, verifying feasibility and potential effectiveness. |
| 9 | Zhang et al.(17) | 2019 | USA | Depression, Anxiety Disorders | APP | 301 | 8 weeks | Baseline, 8w | Used mixed methods (qualitative content analysis & PCA) to assess the impact of three specific usage behaviors ("Learn", "Set Goals", "Self-Track") within the IntelliCare mental health app suite on symptoms; "Self-Track" significantly reduced depressive symptoms. |
| 10 | Yap et al.(18) | 2019 | Australia | Depression, Anxiety Disorders | Web | 332 | 52 weeks | Baseline, 12w, 52w | Used an RCT to evaluate the medium-term (12-month) effect of a tailored web-based parenting intervention (PiP) on improving parent-reported risk/protective parenting factors and adolescent depression/anxiety symptoms. |
| 11 | Sankaran et al.(19) | 2019 | Belgium | Coronary Artery Disease | APP | 28 | 16 weeks | Baseline, 8w, 16w | Used a cross-over multidisciplinary design, based on persuasive design principles and personalization, to evaluate the HeartHab app's effect on motivation, physical activity, quality of life, and risk factors in CAD patients. |
| 12 | Pelle et al.(20) | 2019 | Netherlands, Germany | Knee/Hip Osteoarthritis | APP | 483 | 26 weeks | Baseline, 12w, 26w | Developed the dr. Bart app integrated with machine learning based on behavior change models, evaluating its effect on reducing secondary healthcare use, improving self-management (PAM-13), health-related quality of life, and exploring differences between Dutch and German users in app usage, usability, and outcomes. |
| 13 | Coorey et al.(21) | 2019 | Australia | Cardiovascular Disease | Web | 397 | 52 weeks | Baseline, 52w | Developed a consumer web portal (CONNECT) integrated with primary care Electronic Health Records (EHR) based on the PSD framework, using mixed methods to evaluate its persuasive design features. |
| 14 | Teeriniemi et al.(22) | 2018 | Finland | Obesity | Web | 532 | 104 weeks | Baseline, 52w, 104w | Based on CBT and PSD, developed and evaluated a web-based HBCSS, alone or combined with varying intensity lifestyle interventions (CBT group/self-help guidance), for long-term (2-year) weight loss and maintenance in overweight/obese adults. |
| 15 | Klaassen et al.(23) | 2018 | Netherlands | Diabetes | APP, Web, Wearables | 21 | 6-8 weeks | Baseline, Post-Intervention | Based on the PERGAMON framework, used mixed methods to evaluate the impact of a platform integrating sensors, a virtual coach, and serious games on usability, user experience, and self-management motivation in adolescents with diabetes. |
| 16 | Karppinen et al.(24) | 2018 | Finland | Metabolic Syndrome (e.g., Diabetes, Obesity) | Web | 43 | 52 weeks | Baseline, 12w, 25-27w, 30-52w | Based on the PSD framework and habit formation stage theory, used qualitative methods to evaluate the impact of the web-based BCSS (Onnikka) on habit formation and weight management in high-risk individuals. |
| 17 | Bartlett et al.(25) | 2017 | UK | Chronic Obstructive Pulmonary Disease (COPD) | APP | 121 | Not Mentioned | Baseline, Unspecified | Used mixed methods to investigate COPD patients' perceptions of mobile apps with different design principles. |
| 18 | Kelders et al.(26) | 2015 | Netherlands | Depression | Web | 239 | 12 weeks | Baseline, 12w, 24w | Used an RCT to compare the effects of human-supported vs. automated-supported web platforms for patients with mild-to-moderate depression. |
| 19 | Salvi et al.(27) | 2015 | Spain, Germany, UK | Coronary Artery Disease, Post-MI Rehabilitation | Wearable Device, APP, Web | 55 | 26 weeks | Baseline, 5w, 21w, 26w | Used an RCT to evaluate the impact of the mobile health system (GEx) on education level, exercise adherence, and long-term exercise habits in CAD (post-MI) patients during cardiac rehabilitation. |
| 20 | Ahtinen et al.(28) | 2013 | Finland | Psychological Issues (e.g., Depression, Anxiety) | APP | 15 | 4 weeks | Baseline, 1w, 4w | Evaluated the effect of an Acceptance and Commitment Therapy (ACT)-based mobile app ("Oiva") on reducing work-related stress and improving life satisfaction. |

**Supplementary Table 3.**Frequency and percentage of persuasive principles applied in the 20 included studies.

| Category | Persuasive Principle | De Oliveira et al.[9] | Turkkila et al.[10] | Guracho et al.[11] | Bente et al.[12] | Signorelli et al.[13] | Francis et al.[14] | Yin et al.[15] | Daud et al.[16] | Zhang et al.[17] | Yap et al.[18] | Sankaran et al.[19] | | Pelle et al.[20] | | Coorey et al.[21] | | Teeriniemi et al.[22] | | Klaassen et al.[23] | | Karppinen et al.[24] | | Bartlett et al.[25] | | Kelders et al.[26] | | Salvi et al.[27] | | Ahtinen et al.[28] | | Frequency | | Percentage (%) | |
| --- | --- | --- | --- | --- | --- | --- | --- | --- | --- | --- | --- | --- | --- | --- | --- | --- | --- | --- | --- | --- | --- | --- | --- | --- | --- | --- | --- | --- | --- | --- | --- | --- | --- | --- | --- |
| Primary Task Support | Reduction | * | * | * |  |  | * | * |  |  |  |  |  | | * | | * | | * | | * | |  | |  | |  | | * | | 10 | | 50.00% | |  |
|  | Tunneling |  | * | * | * | * |  | * |  |  | * |  |  | | * | | * | | * | | * | | * | |  | |  | | * | | 12 | | 60.00% | |  |
|  | Tailoring | * | * | * | * |  |  |  |  |  | * | * |  | |  | | * | |  | | * | |  | | * | |  | |  | | 9 | | 45.00% | |  |
|  | Personalization |  |  |  | * | * |  |  |  |  | * | * |  | | * | |  | |  | |  | | * | | * | |  | |  | | 7 | | 35.00% | |  |
|  | Self-monitoring | * | * | * | * | * | * | * | * | * | * | * | * | | * | |  | |  | | * | | * | |  | | * | |  | | 16 | | 80.00% | |  |
|  | Simulation |  |  | * | * |  |  |  |  |  |  | * |  | | * | |  | |  | |  | |  | | * | | * | |  | | 6 | | 30.00% | |  |
|  | Rehearsal | * |  | * |  |  |  |  |  |  | * |  |  | |  | |  | |  | |  | |  | | * | | * | |  | | 5 | | 25.00% | |  |
| Dialogue Support | Praise | * | * | * | * | * |  |  |  |  | * | * |  | | * | | * | | * | | * | | * | | * | |  | |  | | 13 | | 65.00% | |  |
|  | Rewards |  |  |  | * |  | * | * | * |  |  |  | * | | * | |  | | * | |  | | * | |  | | * | | * | | 9 | | 45.00% | |  |
|  | Reminders | * | * | * | * | * | * | * |  | * | * | * | * | | * | | * | | * | | * | | * | | * | | * | |  | | 18 | | 90.00% | |  |
|  | Suggestion |  | * | * | * | * |  | * | * |  | * | * |  | | * | |  | | * | | * | | * | | * | | * | |  | | 14 | | 70.00% | |  |
|  | Similarity |  |  |  |  |  |  |  |  |  |  |  |  | | * | |  | | * | |  | |  | |  | |  | |  | | 2 | | 10.00% | |  |
|  | Liking |  | * | * |  |  |  |  |  |  |  |  |  | |  | |  | |  | | * | |  | | * | |  | |  | | 4 | | 20.00% | |  |
|  | Social Role |  |  | * | * | * |  |  | * |  | * | * |  | | * | |  | | * | |  | | * | | * | |  | |  | | 9 | | 45.00% | |  |
| System Credibility Support | Trustworthiness | * |  | * |  |  |  |  |  |  |  |  |  | | * | |  | |  | |  | |  | |  | | * | | * | | 5 | | 25.00% | |  |
|  | Expertise | * |  | * | * |  |  |  |  |  | * | * |  | | * | |  | |  | |  | |  | |  | |  | | * | | 7 | | 35.00% | |  |
|  | Surface Credibility |  |  |  |  |  |  |  |  |  |  |  |  | |  | |  | |  | |  | |  | |  | |  | |  | | 0 | | 0.00% | |  |
|  | Real-World Feel |  |  | * |  |  |  |  |  |  |  |  |  | |  | |  | |  | |  | |  | |  | |  | |  | | 1 | | 5.00% | |  |
|  | Authority |  |  |  | * |  |  | * | * |  |  | * |  | | * | |  | |  | |  | | * | |  | |  | |  | | 6 | | 30.00% | |  |
|  | 3rd-Party Endorsed |  |  | * |  |  |  |  |  |  |  |  |  | |  | |  | |  | |  | |  | |  | |  | |  | | 1 | | 5.00% | |  |
|  | Verifiability |  | * | * |  |  |  |  |  |  |  |  |  | | * | |  | |  | | * | |  | |  | |  | |  | | 4 | | 20.00% | |  |
| Social Support | Social Learning |  | * |  |  |  |  |  |  |  |  |  |  | |  | |  | |  | | * | | * | |  | |  | |  | | 3 | | 15.00% | |  |
|  | Social Comparison |  |  |  |  |  | * |  |  |  |  |  |  | | * | |  | |  | |  | | * | |  | |  | |  | | 3 | | 15.00% | |  |
|  | Normative Influence |  |  |  |  |  |  |  |  |  |  |  |  | | * | |  | |  | |  | |  | |  | |  | |  | | 1 | | 5.00% | |  |
|  | Social Facilitation |  | * |  |  |  |  |  |  |  |  |  |  | |  | |  | |  | | * | |  | |  | |  | |  | | 2 | | 10.00% | |  |
|  | Cooperation |  |  | * | * |  |  |  |  |  |  |  |  | |  | |  | |  | |  | | * | |  | |  | |  | | 3 | | 15.00% | |  |
|  | Competition |  |  |  | * |  |  |  |  |  |  |  |  | |  | |  | |  | |  | | * | |  | |  | |  | | 2 | | 10.00% | |  |
|  | Recognition |  |  |  |  |  |  |  |  |  |  |  |  | |  | |  | |  | |  | | * | |  | |  | |  | | 1 | | 5.00% | |  |
